# A Src-Like Inactive Conformation in the Abl Tyrosine Kinase Domain

**DOI:** 10.1371/journal.pbio.0040144

**Published:** 2006-05-02

**Authors:** Nicholas M Levinson, Olga Kuchment, Kui Shen, Matthew A Young, Michael Koldobskiy, Martin Karplus, Philip A Cole, John Kuriyan

**Affiliations:** **1**Department of Molecular and Cell Biology, University of California Berkeley, Berkeley, California, United States of America; **2**Department of Chemistry, University of California Berkeley, Berkeley, California, United States of America; **3**Howard Hughes Medical Institute, University of California Berkeley, Berkeley, California, United States of America; **4**Department of Pharmacology, Johns Hopkins University School of Medicine, Baltimore, Maryland, United States of America; **5**Department of Chemistry and Chemical Biology, Harvard University, Cambridge, Massachusetts, United States of America; **6**Physical Biosciences Division, Lawrence Berkeley National Laboratory, Berkeley, California, United States of America; Samuel Lunenfeld Research InstituteCanada

## Abstract

The improper activation of the Abl tyrosine kinase results in chronic myeloid leukemia (CML). The recognition of an inactive conformation of Abl, in which a catalytically important Asp-Phe-Gly (DFG) motif is flipped by approximately 180° with respect to the active conformation, underlies the specificity of the cancer drug imatinib, which is used to treat CML. The DFG motif is not flipped in crystal structures of inactive forms of the closely related Src kinases, and imatinib does not inhibit c-Src. We present a structure of the kinase domain of Abl, determined in complex with an ATP–peptide conjugate, in which the protein adopts an inactive conformation that resembles closely that of the Src kinases. An interesting aspect of the Src-like inactive structure, suggested by molecular dynamics simulations and additional crystal structures, is the presence of features that might facilitate the flip of the DFG motif by providing room for the phenylalanine to move and by coordinating the aspartate side chain as it leaves the active site. One class of mutations in BCR–Abl that confers resistance to imatinib appears more likely to destabilize the inactive Src-like conformation than the active or imatinib-bound conformations. Our results suggest that interconversion between distinctly different inactive conformations is a characteristic feature of the Abl kinase domain.

## Abstract 2

Src- and Abl-kinases are thought to have distinct inactive conformations, accounting for differential responses to the drug imantinib, used to treat chronic myeloid leukemia. Here, however, Abl-kinases are shown to also have Src-like conformational states.

## Introduction

The evolution of specialized regulatory mechanisms in protein kinases is reflected in the range of distinct inactive conformations of kinase domains from different subfamilies [ 1, 2]. This array of alternative inactive states provides opportunities for the development of selective kinase inhibitors, as exemplified by the success of imatinib (Gleevec, Glivec; Novartis) in blocking the activity of BCR–Abl [ 3] and its efficacy in the treatment of chronic myelogenous leukemia. BCR–Abl is a constitutively activated form of the nonreceptor tyrosine kinase c-Abl. The kinase domains of BCR–Abl and c-Abl are identical in sequence, and we shall use “Abl” to refer jointly to BCR–Abl and c-Abl in this paper. Structural differences between the inactive conformations of tyrosine kinase domains of Abl and its close relative c-Src suggest possible explanations for why imatinib can inhibit the kinase domain of Abl but not that of c-Src [ 3, 4].

**Figure 1:**
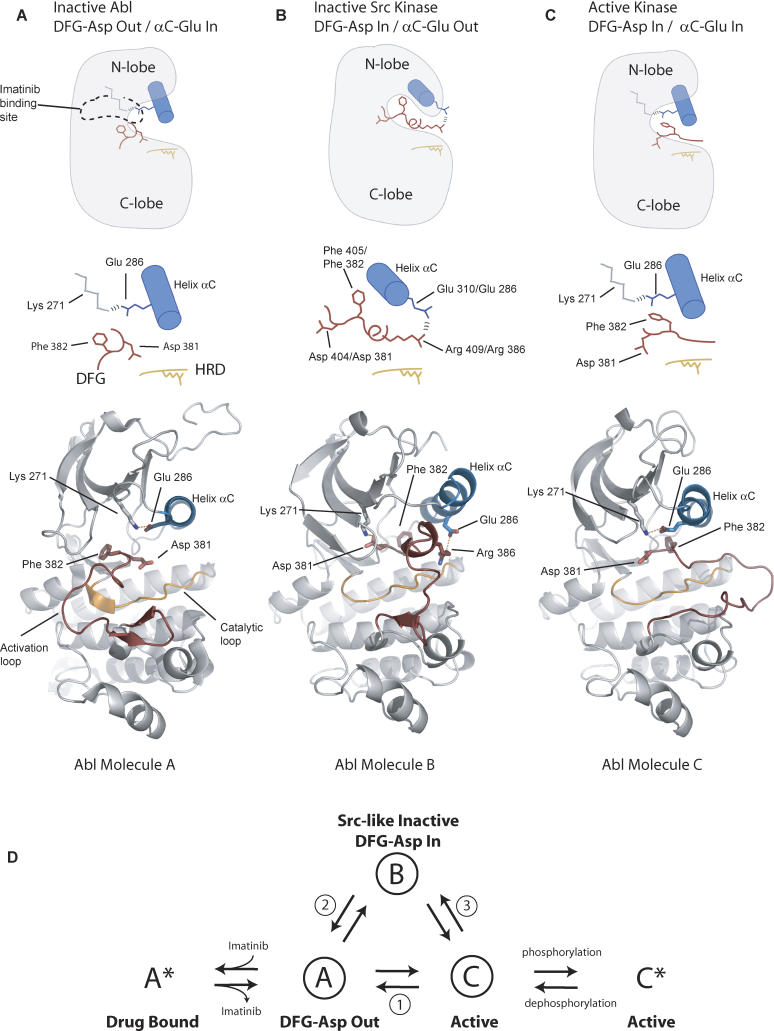
Distinct States of the c-Abl and c-Src Kinase Domains Three key kinase domain conformations considered at length in the text are shown in (A–C). At the top, a schematic representation of each state and an enlarged schematic are shown, detailing the conformations of the DFG motif (red) and helix αC (blue). Below the schematics the crystal structure of each conformation is shown. The activation loop is colored red, helix αC blue, and the catalytic loop orange. (A) The conformation of inactive c-Abl, bound to imatinib (molecule A). (B) The conformation of the inactive Src family kinases. This conformation is now seen in Abl as well (molecule B, structure 1). Both the c-Src and Abl numbering are indicated. (C) Active Abl (molecule C, structure 2). (D) In cells the active conformation of Abl undergoes rapid autophosphorylation that is expected to trap the protein in the active conformation (indicated as C*). Similarly, imatinib only binds to Abl when the kinase domain adopts conformation A and forms a stable complex with the protein (A*). The interconversion between the different states of Abl is shown in the context of this competition.

The structural analysis of small molecule inhibitors bound to p38-Map kinase [ 5] and imatinib bound to Abl [ 4] have illustrated how the recognition of inactive conformations of kinase domains can lead to specificity in kinase inhibition. These and subsequent studies have led to the concept that the inactive states of kinases, which have varied structures because they are not constrained by the necessity of catalyzing the phospho-transfer reaction, can provide additional opportunities for specificity in inhibitor interactions with the highly conserved kinase active sites. In this paper we present the results of new crystallographic studies on the kinase domain of Abl, which reveal a hitherto unappreciated degree of conformational variability in this kinase domain.

c-Src and c-Abl are closely related nonreceptor tyrosine kinases whose catalytic activities are controlled by their Src homology domains SH2 and SH3 [ 6]. In structures of inactive Src kinases, the SH2 and SH3 domains stabilize a conformation of the kinase domain in which a prominent α-helix (helix αC) in the N-terminal lobe (N-lobe) is swung out of the active site, breaking an ion pair between two conserved residues, Glu 310 in helix αC and Lys 295 in the β-sheet of the N-lobe [ 7, 8]. The N-lobe closes down over the C-terminal lobe (C-lobe) of the kinase domain. A centrally located “activation loop” also changes structure, switching from an open and extended conformation in the active state to a more closed conformation in the inactive state. A conserved and catalytically crucial Asp-Phe-Gly (DFG) motif [ 2] is located at the base of the activation loop. The portion of the activation loop that immediately follows the DFG motif forms a helical turn that packs against the outwardly displaced helix αC ( Figure 1). The rest of the activation loop is flexible and is not always visualized in the same conformation in inactive Src kinases.

We refer to the mode of inactivation used by the Src family kinases as “αC-Glu Out,” because the breaking of the conserved ion pair and the outward movement of helix αC are distinctive features of this conformation. The Src-like inactive conformation is illustrated in Figure 1B and is contrasted to the conformation of an active kinase domain shown in Figure 1C. The conformation of the active kinase domain is usually stabilized by phosphorylation of the activation loop.

The SH2 and SH3 domains of inactive c-Abl dock onto the kinase domain in a slightly different manner than in the inactive Src kinases, and this difference is correlated with differences in the structures of the inactive kinase domains of Abl and Src [ 9, 10]. Helix αC is rotated inward in inactive c-Abl, and the conserved ion pair between Lys 271 and Glu 286 (Abl 1a numbering) is formed (“αC-Glu In” in contrast to “αC-Glu Out” in c-Src). The orientation of the two lobes of the kinase domain of inactive c-Abl is essentially the same as in active kinases and is more open than inactive c-Src. The critical conformational change in inactive c-Abl with respect to the active form involves an approximately 180° rotation in the backbone torsion angle ϕ of Asp 381 of the DFG motif, which removes the side chain of Asp 381 from the site of Mg ^2+^ coordination (“DFG-Asp Out,” in contrast to the conformation in active kinases, which we call “DFG-Asp In”). The region of the activation loop that is C-terminal to the DFG motif blocks substrate binding by mimicking the conformation of a kinase substrate peptide [ 4, 11, 12]. Because the activation loop is clearly flexible, we view the DFG flip as the defining inactivating transition of the Abl kinase domain ( Figure 1A).

The flipped conformation of the DFG motif (DFG-Asp Out) is required for high-affinity imatinib binding, and the Src family inactive conformation (αC-Glu Out and DFG-Asp In) is not bound with high affinity by imatinib. The crystal structures of the tyrosine kinase domains of the insulin receptor, c-Kit (with and without imatinib bound), Flt3, Csk, and the Ser/Thr kinases Raf and p38, have revealed that many different kinases, with no specific relationship to Abl, can adopt conformations in which the DFG motif is flipped [ 12– 17]. Kinase inhibitors that recognize the flipped DFG conformation are promising drug development leads. Imatinib itself is already being used successfully in the treatment of gastrointestinal stromal tumors and myeloproliferative diseases caused by the activation of the tyrosine kinases c-Kit and PDGFR [ 18– 20].

The key attributes of the inactive conformation of the Src kinases are also seen in the cyclin-free inactive form of CDK2 [ 21], the kinase domain of the epidermal growth factor receptor [ 22], and the kinase domain of GCN2, a protein kinase involved in the cellular stress response [ 23]. Thus, the inactive conformations of c-Abl and c-Src appear to be representative of two disparate groups of kinases that, in their inactive forms, either have the DFG motif flipped (DFG-Asp Out) or have helix αC rotated out of the active site (αC-Glu Out).

The work presented here concerns structures of the isolated kinase domain of Abl, determined in the absence of the SH2 and SH3 domains or other regulatory domains. The presence of these additional domains significantly influences the catalytic properties of full-length c-Abl and BCR–Abl. The BCR segment of BCR–Abl, for example, induces oligomerization of the protein, which facilitates the trans-autophosphorylation of the Abl kinase domain [ 24]. Autophosphorylation, in turn, is believed to stabilize the kinase domain in the active conformation. Nevertheless, understanding the conformational landscape of the isolated and unphosphorylated kinase domain is of interest because the presence of additional domains, or posttranslational modifications, such as phosphorylation, is likely to modulate the degree to which conformations that are accessible to the isolated kinase domain are populated.

In the first part of this paper we present crystal structures of the Abl kinase domain bound to substrate-mimicking ATP analog–peptide conjugates that represent four distinct conformations of the unphosphorylated kinase domain. Unexpectedly, one of these structures of the Abl kinase domain has a striking similarity to the conformation of inactive Src kinases (DFG-Asp In, αC-Glu Out). This is the first time that the same protein has been seen to adopt both the DFG-Asp Out and the αC-Glu Out inactive conformations. The other structures include active and various inactive conformations that suggest how the kinase domain might convert between the αC-Glu In and αC-Glu Out conformations. In the second part of the paper we present a speculation, based on computer simulations and the new crystal structures, regarding a potential role for the Src-like inactive structure in facilitating the DFG flip. In the last part of the paper we identify a class of imatinib resistance mutations in the BCR–Abl kinase domain whose locations in the structure suggest that destabilization of the Src-like inactive structure might impact the binding of imatinib to Abl.

## Results/Discussion

### Crystal Structures

In this paper we discuss five distinct conformations of the kinase domain of Abl, based on four new crystal structures as well as the previously published structure of the Abl:imatinib complex ( Table 1). The protein samples used for crystallization consisted of the isolated kinase domain that was not phosphorylated on the activation loop, and we assume that increased flexibility of the protein under these conditions is responsible for the isolation of multiple distinct conformations. The crystallographic data and refinement statistics are noted in Table S1.

**Table 1:**
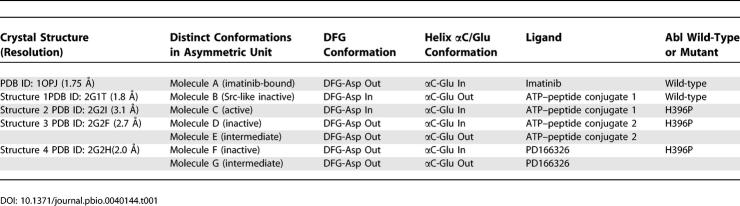
Crystal Structures of the Kinase Domain of Abl

The first of these conformations is that of the kinase domain bound to imatinib, determined previously [ 9], and is denoted molecule A ( Table 1 and Figure 1A). The remaining conformations are derived from three different crystal forms obtained in the presence of ATP analog–peptide conjugates [ 25]. These bisubstrate analogs inhibit the activity of the Abl kinase domain with K _i_ values of approximately 1 μM (see Protocol S1 for a description of the kinetic characterization). In the first of our new structures ( Table 1, molecule B; and Figure 1B), the kinase domain of Abl assumes a conformation that is strikingly similar to that of the inactive Src kinases (DFG-Asp In/αC-Glu Out). We obtained two additional crystal forms, representing three different conformations of the kinase domain, using a construct of Abl that contains a patient-derived imatinib resistance mutation in the activation loop, His 396 Pro (H396P).

One crystal form of the H396P mutant has Abl in an active conformation ( Table 1, molecule C). These crystals diffracted X-rays weakly, and the structure was solved to 3.1 Å resolution. Molecule C is shown in Figure 1C and is very similar to the crystal structure of the kinase domain of Abl H396P bound to the small molecule inhibitor VX-680 [ 26], which has provided a high-resolution (2.0 Å) model of the active conformation of the Abl kinase domain [ 27]. The isolation of the same conformation in the presence of an ATP analog–peptide conjugate demonstrates that these substrate analogs are compatible with the active form of the kinase domain.

The other crystal form of the H396P mutant has two molecules in the asymmetric unit that are in different conformations. The first of these molecules (molecule D) is in an inactive conformation with a flipped DFG motif (DFG-Asp Out/αC-Glu In) despite the presence of the ATP analog in the nucleotide-binding site. Previously, DFG-Asp Out conformations of Abl have only been seen in the presence of high-affinity kinase inhibitors such as imatinib and PD166326. The appearance of the DFG-Asp Out conformation in the presence of a nucleotide analog shows that this conformation is an accessible state of the kinase domain of Abl and is not induced only by the binding of high-affinity kinase inhibitors. Because of the similarity to previously reported structures of Abl [ 9, 28], we do not consider this conformation further.

The second molecule (molecule E) in this crystal form is in an intermediate conformation, with helix αC partially swung out of the active site and with disruption of the conserved ion pair between Glu 286 and Lys 271 (DFG-Asp Out/αC-Glu Out). The same crystal form was also obtained with the small molecule inhibitor PD166326 [ 29]. Crystals obtained with PD166326 are isomorphous to those obtained with the peptide–ATP conjugate, but diffract X-rays to higher resolution (see Materials and Methods). The resulting models are very similar, with differences limited to local changes that are a consequence of the different ligands. The conformation of Abl represented by molecules E and G suggests a mechanism by which Abl can interconvert between αC-Glu In and αC-Glu Out conformations.

### A Structure of the Abl Kinase Domain That Resembles Inactive Src Kinases

**Figure 2:**
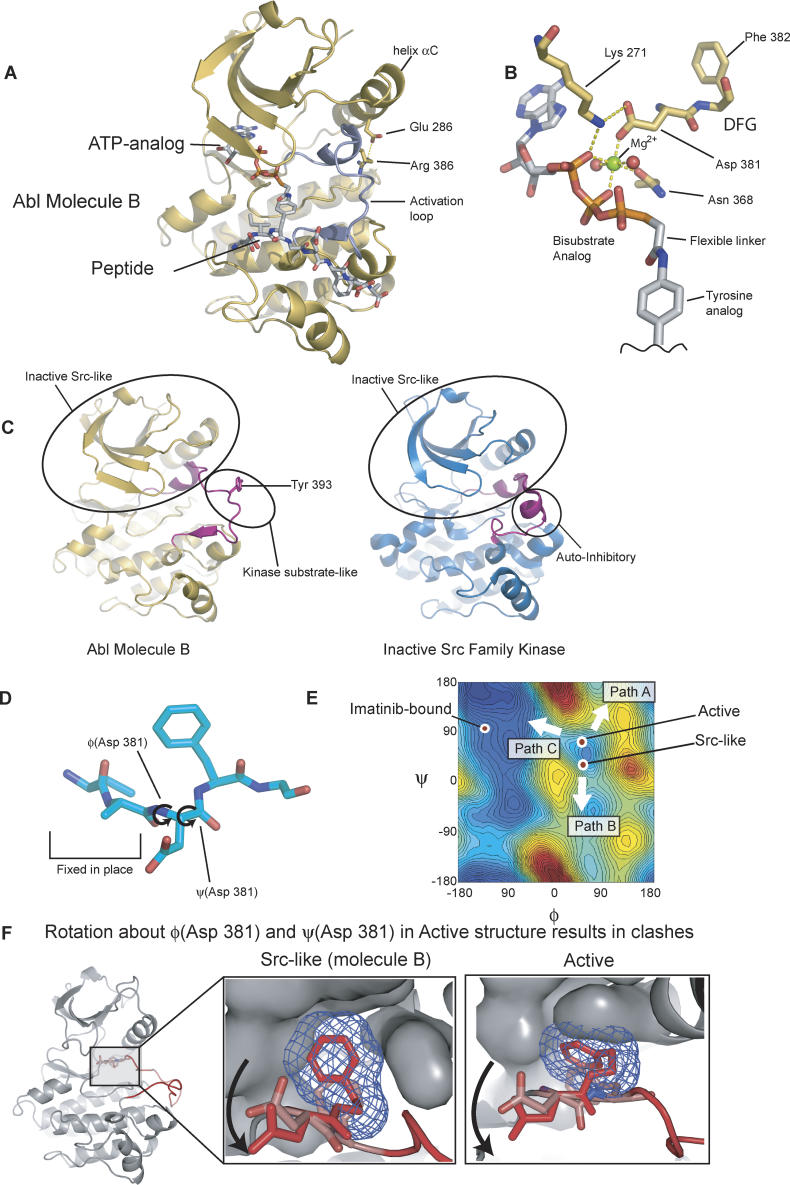
Molecule B Closely Resembles the Structure of the Inactive Src Kinases (A) The kinase domain of Abl (molecule B) is shown as a cartoon with the bisubstrate analog inhibitor depicted as a stick model. (B) Structure of the ATP–peptide conjugate at the active site. (C) Comparison between molecule B (left) and the structure of a Src-family kinase (right). (D) Backbone torsion angles of Asp 381 of the DFG motif. (E) The backbone of Asp 381 can start to move from DFG-In to the DFG-Out conformations by three paths in torsion angle space [ 32]. (F) Illustration of rotations about ϕ(Asp 381) and ψ(Asp 381) in the Src-like inactive versus the active structures of Abl.

The structure of molecule B is shown in Figure 2A. The peptide portion of the substrate analog is bound to the activation loop and the C-lobe of the kinase domain essentially as seen previously in crystal structures of the insulin receptor kinase domain [ 11, 30], except that the activation loop and the bound peptide have rotated outward as a unit with respect to the center of the kinase domain ( Figure 2A and Figure S1).

The conformation of molecule B is dramatically different from all other structures of Abl determined to date, all of which adopt the αC-Glu In conformation. In molecule B, helix αC is swung out of the active site (αC-Glu Out), a conformational change corresponding to an outward rotation of 34° about the helix axis and a 31° pivot of the helix axis with respect to the conformation of helix αC in active Abl ( Table 1, molecule C; and Figure 1C). As a result of the movement of the helix, the conserved salt bridge between Lys 271 and Glu 286 is broken. Both of these charged residues form new ion-pairing interactions.

The side chain of Glu 286, which is surface-exposed because of the rotation of helix αC, forms an ion pair with Arg 386 within the activation loop ( Figure 1A). The side chain of Lys 271 forms a salt bridge with the side chain of Asp 381 of the DFG motif in molecule B. As a consequence, the side chain of Asp 381 is moved relative to its position in active structures. The coordinated Mg ^2+^ ion is also displaced and bound between the α and β phosphates of ATP, and not between the β and γ phosphates ( Figure 2B).

The region of the activation loop immediately C-terminal to the DFG motif in molecule B forms a single turn of a 3 _10_ helix that packs against helix αC, forming an extensive hydrophobic interface that stabilizes the αC-Glu Out conformation ( Figure 2A; the activation loop is colored blue). The DFG motif and the leucine residue immediately following a canonical type II β turn [ 31], with Asp 381 occupying the first position in the turn.

Abl molecule B closely resembles the inactive Src kinases ( Figure 2C). Alignment of Abl molecule B (residues 246 to 500, excluding residues 381 to 402 in the activation loop, see below) with the corresponding regions of c-Src [ 7] and Hck [ 8] yields root mean square (r.m.s.) deviations of 1.17 Å and 0.99 Å, respectively, for Cα atoms. The major difference between the structures of Src kinases and the structure of Abl molecule B concerns the central region of the activation loop, residues 389 to 402 in Abl ( Figure 2C). In Abl, this region of the activation loop adopts a conformation in which Tyr 393 is pointed outward and appears to be presented for phosphorylation by another kinase molecule.

### A Speculation Regarding DFG Flips and Src-Like Inactive Conformations, Based on the Ramachandran Diagram

The structure of molecule B indicates, for the first time, that a Src-like inactive conformation is accessible to Abl ( Figure 1B). This led us to ask whether there is a particular functional significance for this conformation. In addition to a possible autoinhibitory role as a stable form of the unphosphorylated kinase domain, certain features of molecule B suggested to us that a Src-like inactive conformation may function as an intermediate conformation that facilitates the DFG flip. The DFG motif is located at the base of the activation loop, in an environment that is tightly packed in the active conformation. A salient feature in both inactive Src and Abl molecule B (Src-like inactive) is the presence of a sizable internal cavity around the DFG motif that is created by the outward rotation of helix αC [ 8]. This cavity is an enlargement of the ATP-binding pocket, and its presence results in the environment of the DFG motif being considerably less hindered sterically in the Src-like inactive conformation than in the active conformation of protein kinases.

The backbone torsion angles involved in the DFG-Asp In/Out transition are shown in Figure 2D. The torsion angles of Asp 381 are very similar in the active (ϕ = 48.0°, ψ = 83.2°) and Src-like inactive conformations of Abl (ϕ = 49.6°, ψ = 36.4°), but distinct in the DFG-flipped conformation (ϕ = −152.9°, ψ = 98.6°). A Ramachandran diagram based on free energy calculations on the isolated alanine dipeptide in aqueous solution [ 32] shows that there are three low-energy paths in backbone torsion angle space for the ϕ and ψ torsion angles of Asp 381 by which the aspartate residue can begin the transition from the active or the Src-like conformation toward the DFG-flipped conformation ( Figure 2E). The most direct route in backbone torsion angle space for this transition involves decreasing the value of ϕ while increasing the value of ψ (Path C in Figure 2E). Such a coupled set of torsion angle changes immediately drives the side chain of Phe 382 into the C-lobe and cannot be counteracted by changes in any other downstream torsion angles. Path C therefore seems unlikely.

The two other low-energy paths, A and B, when coupled to backbone rotations in Phe 382, are nearly equivalent in terms of the movements of the side chains of Asp 381 and Phe 382 (this point is discussed further below). The initial steps along Path A involve increasing the values of both ϕ and ψ for Asp 381. The consequence of a +30° rotation in both ϕ and ψ for Asp 381 in the structure of active Abl is illustrated in Figure 2F. Asp 381 moves down into the base of the ATP-binding site and away from Lys 271, while Phe 382 moves up toward the ATP-binding pocket, but does not enter it. Further movement of Phe 382 appears to be blocked by side chains of helix αC. If the same set of rotations is made in the context of Abl molecule B (Src-like inactive), there is a striking difference. The side chain of Phe 382 now enters the enlarged ATP-binding cavity, and there is no obvious steric block to its continued movement ( Figure 2F).

This simple analysis suggests that structural features in the Src-like inactive structure might facilitate the DFG flip, but a particularly difficult question concerns the mechanism by which the charged side chain of Asp 381 is stabilized as it moves away from the highly polar environment of the ATP-binding site. To gain more insight into this issue we turned to molecular dynamics simulations of the DFG flip mechanism.

### Targeted Molecular Dynamics Simulations of the DFG-Asp In/Out Transition

The DFG-Asp In/Out transition is unlikely to occur spontaneously in conventional molecular dynamics simulations, which are currently restricted to the nanosecond timescale for proteins the size of the Abl kinase domain [ 33]. We therefore employed a version of the targeted molecular dynamics simulation method [ 34], in which an external force is applied to a selected portion of the protein structure in order to drive a desired conformational transition [ 35]. Here we used targeted molecular dynamics to drive the conformation of the DFG motif from the active conformation (DFG-Asp In) or the Src-like conformation (DFG-Asp In) to the imatinib-bound conformation (DFG-Asp Out). These simulations explore the transitions denoted 1 and 2 in Figure 1D.

The targeted molecular dynamics method drives a selected portion of the simulated structure to be at a desired r.m.s. distance from a target structure. The entire kinase domain as well as the surrounding box of water molecules is allowed to move in response to the driving force, which is applied directly to only a portion of the simulated system. The desired r.m.s. distance is decreased gradually during the simulation so that, eventually, the simulated structure is driven to be close to the target structure. The restraint energy, E _restraint_, is given by



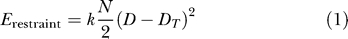



where *k* is force constant, *N* is the number of atoms included in the restraint, *D* is the current mass-weighted r.m.s. distance of the restrained region from the target, and *D _T_* is the desired value of the r.m.s. distance.

For a fully solvated simulation system containing the kinase domain we are able to obtain a 0.2-ns trajectory in about a day using two Intel 3.06 GHz Xeon processors. We carried out multiple trajectories of this length (86 in total) to gauge the effects of parameters such as the force constant and the atoms in the restraint set. A few simulations (12 in total) extended for longer times (1–4.5 ns). The essential features of the transitions are the same in the long and the short trajectories.

**Figure 3:**
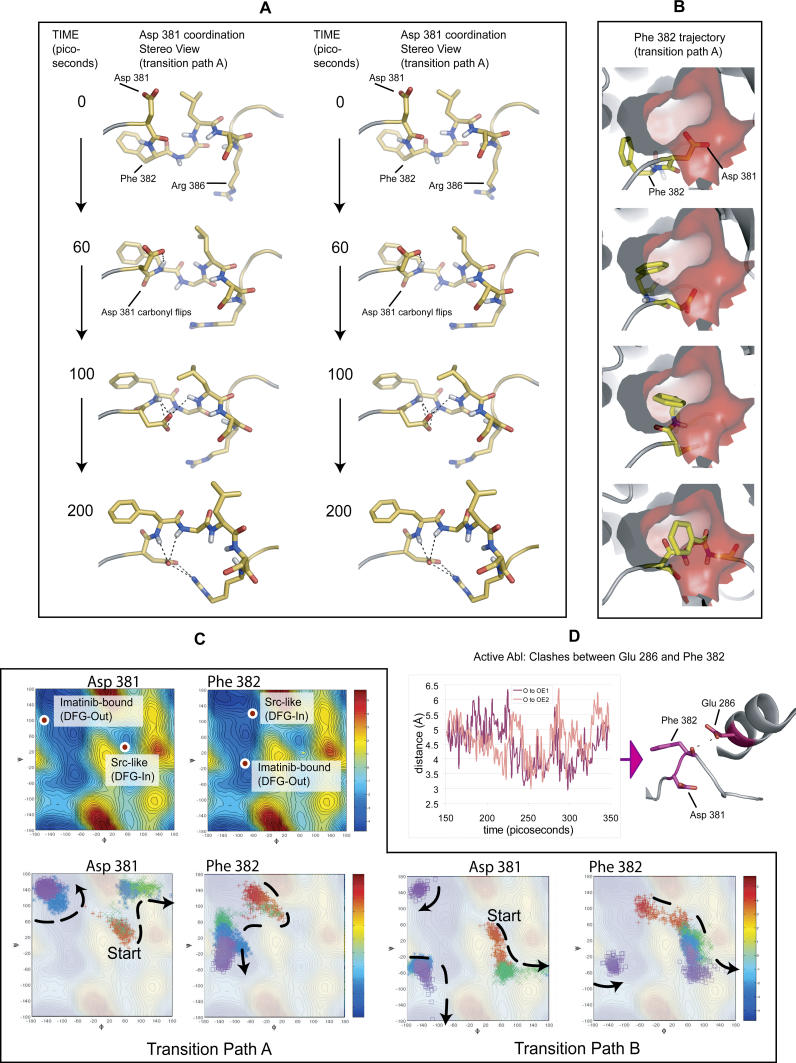
Targeted Molecular Dynamics Simulations Suggest a Path for DFG Flipping (A) Coordination of Asp 381 during the DFG flip, shown in stereo, starting from the Src-like structure and following transition path A in backbone torsion angle space (see Figure 2E). The coordination of Asp 381 is almost identical for transition path B, except that the carbonyl of Asp 381 flips in the other direction (see Figure S3). (B) The side chain of Phe 382 passing through a hydrophobic pocket in the Src-like inactive molecule B. (C) The paths of Asp 381 and Phe 382 in backbone torsion angle space for two trajectories starting with the Src-like inactive structure. Shown in red are the torsion angles seen in the first quarter of a TMD time series, in green, the second quarter of the time series, in blue, the third quarter, and in purple, the last quarter. (D) Unfavorable interactions between the backbone carbonyl of Phe 382 and the carboxylate group of Glu 286 during TMD simulations starting from the active structure, where the Glu 286-Lys 271 salt bridge is present.

Simulations were carried out using restraint sets ranging from the entire kinase domain to only four residues, including the Asp 381 and the Phe 382 residues of the DFG motif ( ^379^VADF ^382^). The manner in which the DFG flip occurs is essentially the same in every trajectory ( Figure 3), and most of our analysis focuses on simulations in which the restraint force was applied to the four-residue segment ^379^VADF ^382^. More details regarding the force constants and the restraint sets are given in Protocol S1.

Because of the strong driving forces necessitated by the short simulation times (see Protocol S1 and Figure S2), these simulations cannot be used to obtain meaningful estimates as to whether the DFG flip occurs at a faster rate in transition 1 (active to DFG flipped) or transition 2 (Src-like inactive to DFG flipped, Figure 1D). Such estimates would require calculation of the free energy barriers to the transitions [ 36– 38] with proper consideration of the role of ATP/ADP•Mg ^2+^ (not included in most of the present simulations; see Protocol S1). This is a time-consuming calculation and is left for future investigations. Instead, the highly consistent pathways generated by the simulations are analyzed in terms of the intermediate structures that are present in the trajectories. Coordinates sampled from representative trajectories are available for download as Datasets S1 and S2.

### Phe 382 Uses the Enlarged ATP-Binding Pocket in the Src-Like Inactive Conformation during Simulated DFG Transitions

The simulations reveal two different transition pathways between the Src-like inactive and the imatinib-bound structures ( Figure 3C) that correspond to the two low-energy paths A and B for Asp 381 backbone torsion angles in the Ramachandran diagram ( Figure 2E). Both paths show the side chain of Phe 382 passing through the enlarged ATP-binding cavity found in the Src-like inactive conformation ( Figure 3B); this is in accord with the analysis based on the Ramachandran diagram, described earlier. The differences between the two paths, A and B, are restricted to the direction of the movement of the backbone carbonyl group of Asp 381 (see Figure S3 for an illustration of a path B transition).

Analogous simulations starting with the active structure of the Abl kinase domain (transition 1 in Figure 1D) show that the active structure can also accommodate the DFG flip, but apparently less well than the Src-like structure. The presence of the salt bridge between Glu 286 and Lys 271 impedes the flip of the phenylalanine residue in the DFG motif, as a significant clash occurs between the oxygen atoms of the Glu side chain and the carbonyl group of Phe 382 in the approximately 30 simulations started from the active conformation ( Figure 3D).

### Features in the Src-Like Inactive Structure That Stabilize Asp 381 during Simulated DFG Flips

**Figure 4:**
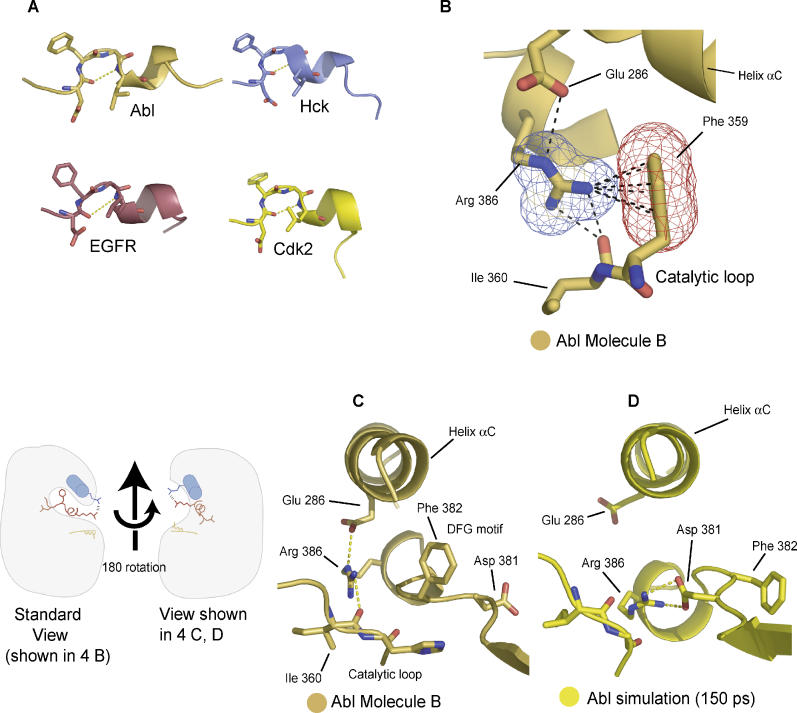
The Helical Turn following the DFG motif in Src-Like Inactive Structures (A) The helical turn in the activation loop of molecule B that immediately follows the DFG motif is a characteristic feature of the Src-like conformation and is conserved in four different kinase families. (B and C) The side chain of Arg 386, presented by the helical turn, forms a hydrogen bond to a backbone carbonyl of Ile 360, a salt bridge with Glu 286, and an amino–aromatic interaction with Phe 359 that positions it for interacting with Asp 381 during the DFG flip. (D) An intermediate structure during one of the TMD simulations, showing the capture of Asp 381 by Arg 386.

All of the simulations initiated from the Src-like inactive structure (transition 2 in Figure 1D) reveal a similar mechanism for how the negative charge of Asp 381 can be coordinated during the transition. The aspartate side chain passes underneath the polypeptide backbone of the DFG motif and forms an extensive hydrogen-bonding network with the backbone amide groups of the next three residues ( Figure 3A). This type of interaction between an Asp side chain and the backbone is commonly seen in crystal structures and is referred to as an Asx turn [ 39]. When this interaction occurs immediately N-terminal to α-helices, the arrangement of the backbone NH groups helps to stabilize the negative charge of the Asp side chain. The 3 _10_ helix immediately following the DFG motif in the molecule B has a corresponding stabilizing interaction with the aspartate of the DFG motif during the flip. This 3 _10_ helix in the activation loop is a characteristic feature of the Src-like inactive conformation, and it has now been observed in four different protein kinase families: Abl, Src, epidermal growth factor receptor, and CDK2 ( Figure 4A).

Asp 381 is also stabilized during the transition by an interaction with Arg 386, a conserved ligand of the phosphorylated tyrosine residue in activated kinases [ 11, 40]. The side chain of Arg 386, which is part of the 3 _10_ helix, forms a salt bridge to the flipped-out Glu 286 in molecule B, an interaction that is also seen in the structures of inactive c-Src and Hck [ 7, 8]. Arg 386 in Abl molecule B is also oriented by an amino–aromatic interaction [ 41] with Phe 359 in the catalytic loop and a hydrogen bond to a backbone carbonyl ( Figure 4B). This orientation of Arg 386 allows it to capture Asp 381 in many of the simulations as it emerges from beneath the backbone of the activation loop ( Figure 3A and Figure 4C and 4D).

There is a qualitative difference between intermediate structures formed in simulations initiated with the Src-like structure (transition 2, Figure 1D) and the active structure (transition 1, Figure 1D). In simulations of both transitions, the first structural changes to occur are the breakage of the ionic interactions between Asp 381 and Lys 271 and the entrance of Phe 382 into the nucleotide-binding pocket, which happen in a concerted manner. In simulations initiated from the Src-like inactive structure, an intermediate is formed in which Asp 381 is coordinated by the backbone amide groups of the next three residues. In the simulations starting from the active structure, Asp 381 is either not coordinated when the ionic interaction breaks, or is coordinated by only one amide group.

The simulations also reveal a potential role for the type II β turn adopted by the ^381^DFGL ^384^ residues of the DFG motif that is a characteristic feature of the Src-like inactive structure but not the active structure. This type II β turn aligns the backbone amide groups of residues Gly 383 and Leu 384 so that they are pointing down toward the C-lobe of the kinase domain, allowing for coordination of the aspartate as it undergoes the transition. A hydrogen bond to a backbone carbonyl in the C-lobe anchors the backbone amide of Gly 383, such that a type II β turn is favored over a type I β turn in the Src-like inactive conformation. The backbone conformation following the DFG motif in the Src-like inactive structure can also be classified as a potential anion-binding site [ 42].

The glycine of the DFG motif is essentially invariant among protein kinases. A role for this residue in the active conformation is suggested by the structure of the serine threonine kinase protein kinase A, in which the presence of glycine in the DFG motif appears to be necessary for proper coordination of Mg ^2+^•ATP [ 43]. Interestingly, type II β turns display a strong preference for glycine at the third position [ 31], suggesting that the Src-like inactive conformation also imposes a requirement for glycine in the DFG motif.

### Additional Structures of Abl Suggest How Helix αC Might Transition to the αC-Glu Out Conformation

The simulations do not address the question of how Glu 286, in helix αC, moves out of the active site to adopt its solvent-exposed position in the Src-like inactive structure. This is an important question because such a transition involves breaking the ion pair between Glu 286 and Lys 271 and is unlikely to happen unless compensating electrostatic interactions are provided ( Table S2). Two crystal structures of Abl reported in this work (molecules E and G) advance our understanding of this question.

**Figure 5:**
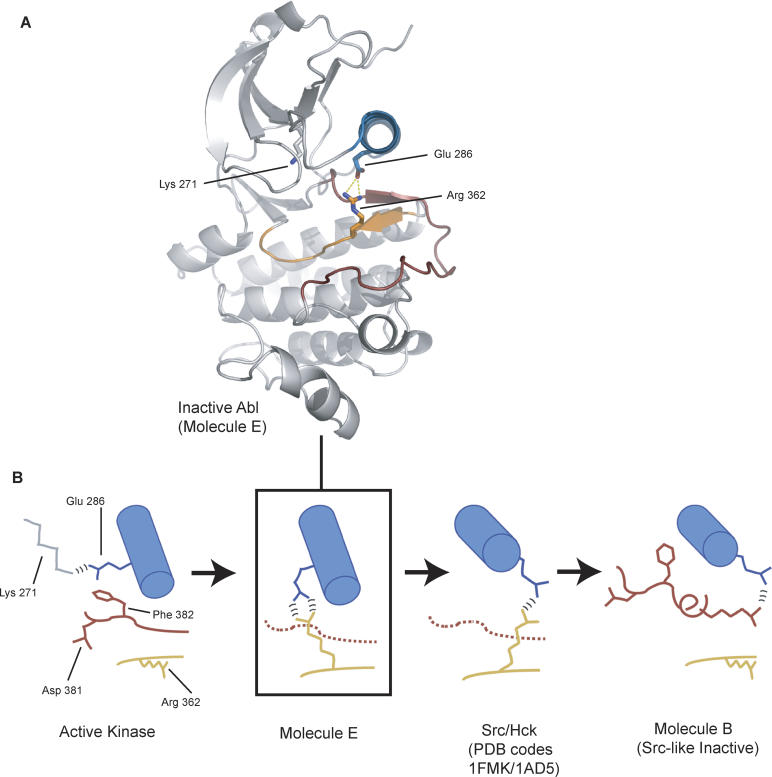
The Structure of Abl in an Intermediate Conformation Suggests a Path for the Transition between the Active and Src-Like Conformations (A) The intermediate structure of Abl (molecule E) is shown, with helix αC shown in blue, the activation loop in red, and the catalytic loop containing Arg 362 in orange. (B) The proposed pathway by which Abl makes the transition to the Src-like conformation.

Molecules E and G were obtained from the same crystal form of Abl and are very similar, except for the presence of either the ATP-peptide analog (molecule E) or the kinase inhibitor PD166326 (molecule G). In these structures, the kinase adopts a conformation intermediate between αC-Glu In and αC-Glu Out: while helix αC is not rotated out of the active site of the kinase, a rotation of the side chain of Glu 286 has occurred, resulting in the breakage of the Glu 286–Lys 271 salt bridge. This interaction is replaced by an ion pair between Glu 286 and Arg 362 in the catalytic loop, part of a conserved His-Arg-Asp (HRD) motif ( Figure 5A). The existence of a salt bridge between Glu 286 and the HRD arginine (Arg 362) in molecules E and G would be expected to offset the energetic cost of breaking the Glu 286–Lys 271 salt bridge. Once the salt bridge is broken, the interface between helix αC and the rest of the N-lobe of the kinase is exclusively hydrophobic. The helix could then rotate relative to the β-sheet of the N-lobe. We propose that this rotation could occur without breaking the stabilizing interaction between Glu 286 and Arg 362 ( Figure 5B).

In support of the idea that Arg 362 in the HRD motif might provide electrostatic stabilization as Glu 286 moves out of the active site, the same ion pair corresponding to Glu 286 and the HRD Arg 362 is seen in the earliest reported structures of the inactive Src kinases, Src and Hck [ 44, 45]. In these structures, helix αC has rotated out while maintaining the Glu-HRD Arg ion pair. In the crystal structure of GCN2, the same ion pair is formed in the context of an αC-Glu Out conformation [ 23]. Thus this ion pair is compatible with both the αC-Glu In and αC-Glu Out conformations. A schematic of the proposed αC-Glu In/Out transition pathway is shown in Figure 5B.

### Potential Relevance for a Src-Like Inactive Structure in Imatinib Resistance

Clinical resistance to imatinib therapy is a growing problem in the treatment of chronic myeloid leukemia [ 46]. Mutations that confer resistance to imatinib have been mapped using patient-derived samples and by using in vitro screens [ 47, 48]. Surprisingly, only a minority of the imatinib resistance mutations result in direct blockage of the drug-binding site. A larger class of mutations appear to act by destabilizing the specific inactive conformation of Abl to which imatinib binds [ 47]. A third class of mutations cannot be rationalized easily in this manner; many of these occur in regions of the structure that have essentially identical conformation in the imatinib-bound and active conformations, or are solvent-exposed. It is unclear how mutations at such sites preferentially destabilize the imatinib-bound conformation, while allowing the kinase to retain catalytic activity.

**Figure 6:**
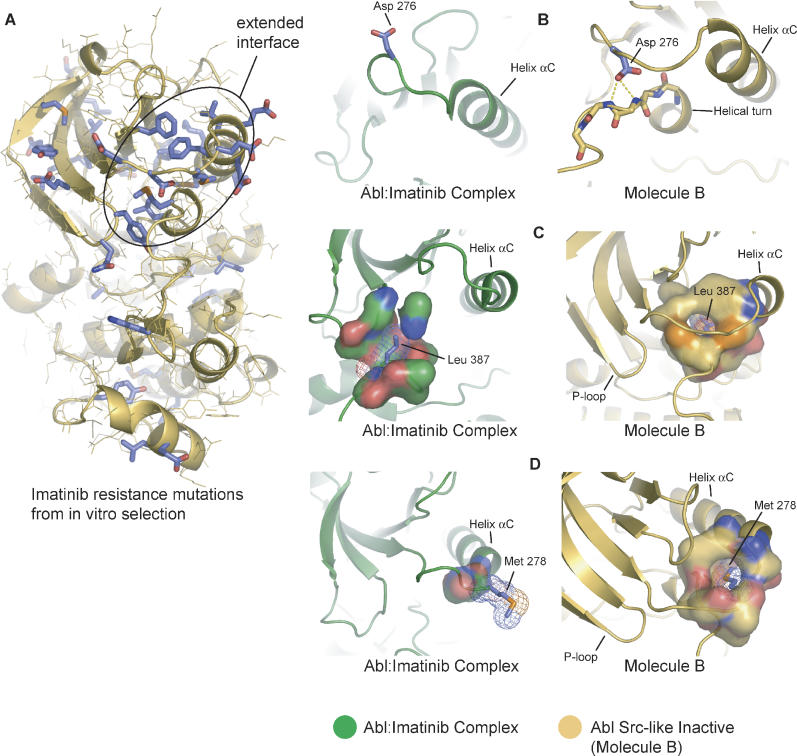
Molecule B Helps Explain Mutations in the Kinase Domain of Abl That Confer Resistance to Imatinib (A) The side chains of residues implicated in imatinib resistance in the Azam et al. study [ 48] are shown in blue in the context of molecule B. A large number of these mutations cluster in the interface between helix αC, the N-lobe, and the helical turn in the activation loop. (B–D) For three of these mutations we have shown the surface for all atoms within 6 Å of the mutated residue in the context of different structures. The Abl:imatinib complex is in green and the Src-like structure (molecule B) in yellow-orange. (B) Asp 276. (C) Leu 387. (D) Met 278.

Notably, a striking number of resistance mutations map to the interface between helix αC and the N-lobe of the kinase ( Figure 6A). This interface is preserved between the active and imatinib-bound structures, but in the Src-like inactive structure (molecule B) the altered orientation of helix αC and the presence of the 3 _10_ helix in the activation loop result in a much larger interface. Mapping the location of the resistance mutations onto molecule B shows that almost every residue at the enlarged αC/N-lobe interface can confer imatinib resistance when mutated ( Figure 6A).

Several of the resistance mutations play a particularly important structural role in the Src-like conformation of Abl, but are of less obvious importance in the imatinib-bound or active conformations. For example, the side chain of Asp 276 makes two strong hydrogen bonds with backbone amide groups in the activation loop immediately following the helical turn in molecule B ( Figure 6B, right). These interactions help anchor the activation loop in the Src-like conformation and would be disrupted by the patient-derived resistance mutation identified at this position (D276G) [ 49] as well as by the D276V mutation isolated in the in vitro screen [ 48]. The importance of similar interactions in the maintenance of the inactive form of c-Src has been demonstrated previously [ 50]. In contrast, Asp 276 is solvent-exposed in the Abl:imatinib complex ( Figure 6B, left) and in the active structure.

Two more examples are the hydrophobic residues Leu 387 and Met 278, which are completely buried in the extended interface between helix αC, the N-lobe, and the helical turn in the activation loop in the Src-like inactive structure (molecule B; Figure 6C and 6D, right). Mutations of Leu 387 have been isolated in the patient-resistance studies (L387F/M), while a mutation of Met 278 (M278L) was identified in the in vitro screen. In the imatinib complex, both Leu 387 and Met 278 are largely solvent-exposed ( Figure 6C and 6D, left). Other mutations are discussed in Protocol S1 and Figure S4.

Activation-loop phosphorylation is expected to stabilize Abl in the active conformation, and imatinib binds to phosphorylated Abl with greatly reduced affinity. Although BCR–Abl is phosphorylated constitutively in cells, tyrosine phosphatases ensure that a dynamic interconversion exists between the phosphorylated and unphosphorylated forms of the protein ( Figure 1D [ 51]), and the continual formation of unphosphorylated BCR–Abl through this interconversion is critical for the efficacy of imatinib [ 4, 28, 51]. Because of the competition between autophosphorylation and imatinib binding, mutations that perturb the balance between the different conformational states of Abl could have a dramatic effect on the efficacy of imatinib.

### Conclusions

The central result of this paper is the discovery that the kinase domain of Abl can readily adopt a conformation that resembles closely that of the inactive Src kinases. This is a surprise, because all previously seen conformations of the inactive forms of the Abl kinase domain have in common a flipped DFG motif (DFG-Asp Out/αC-Glu In), whereas the DFG motif is not flipped in the structures of the inactive Src kinases. The high-affinity binding of imatinib to Abl and its other targets requires a flipped DFG motif. Although c-Src binds to imatinib with low affinity, other Src kinases, such as Lck, bind to imatinib with greater affinity [ 52, 53]. This suggests that just as Abl can adopt a Src-like inactive structure, the Src kinases can, with varying ease, adopt the inactive conformation that is characteristic of Abl.

Our results link, for the first time, Src-like inactive and DFG-flipped inactive conformations in the same protein molecule. Because of the location of the DFG motif deep within the active-site cleft of the kinase, the flipping of the DFG motif is unlikely to occur without significant rearrangements in the rest of the kinase domain. We hypothesize, based on analysis of the structures in combination with targeted molecular dynamics simulations, that the DFG flip might be facilitated by adoption of the Src-like inactive structure. When helix αC is moved out of the active site, the DFG motif is freed from the steric hindrance imposed by the salt bridge between Glu 286 and Lys 271. The side chain of Phe 382 can then move through the sizable hydrophobic pocket created by the outward displacement of helix αC. Throughout the flip, the negative charge of Asp 381 is stabilized by the backbone amide groups of the activation loop and interaction with Arg 386.

The discovery that Abl adopts a Src-like inactive conformation suggests a mechanism by which one class of mutations in the kinase domain of Abl might allow the protein to escape inhibition by imatinib. These mutations map to regions of the kinase domain that undergo major structural changes when the kinase adopts the Src-like inactive conformation. These mutations might affect kinase activity at equilibrium if the Src-like inactive conformation represents a major population of the inactive Abl molecules in solution. Such effects may be difficult to detect in the isolated kinase domain of Abl, in which conformational transitions do not appear to be rate-limiting when compared to substrate binding and release or the chemical step, as evidenced by the fact that phosphorylation does not increase the activity of the isolated kinase domain [ 4]. A previous study of resistance mutations using only the kinase domain of Abl has shown only subtle effects for several resistance mutations [ 54]. It may be important to study the attributes of resistance in the context of BCR–Abl or large constructs of c-Abl.

The DFG motif is conserved across almost every member of the eukaryotic protein kinase superfamily [ 2]. In addition, the surrounding residues that interact with the DFG motif are also highly conserved [ 2]. This makes it challenging to devise experiments that involve mutagenesis of the DFG motif or residues in its immediate environment in such a way that the results could be interpreted without ambiguity. Characterization of the imatinib resistance mutations may provide one experimental route by which to investigate the effect of mutations on the conformational changes in the kinase domain of Abl. Ultimately, however, NMR measurements of structure and dynamics, combined with computer simulations, are likely to be required to establish the relevance of the Src-like inactive structure for the regulation of Abl. In this regard, it is encouraging that preliminary NMR analyses of dynamics in kinase domains are yielding promising results [ 55, 56].

## Materials and Methods

### Bacterial expression of Abl.

The kinase domain of human c-Abl (residues 229–512) was coexpressed with the tyrosine phosphatase YopH in bacteria according to a published protocol [ 27, 57]. The protein was purified by affinity and anion exchange chromatography, and size-exclusion chromatography showed that the protein was monomeric and free of aggregates. The H396P mutation was made using the QuikChange site-directed mutagenesis kit (Stratagene, La Jolla, California, United States) and purified as above. Proteins were concentrated to 10 mg/ml in gel filtration buffer (50 mM Tris-HCl [pH 8.0], 100 mM NaCl, 10% glycerol, and 0.5 mM DTT), snap-frozen in liquid nitrogen, and stored at −80 °C.

### Synthesis of the bisubstrate analog inhibitors.

The peptide portions of the substrate analogs were assembled by solid-phase peptide synthesis using Fmoc chemistry, performed as reported previously [ 25, 30], with minor modifications. The tyrosine of the substrate was replaced by azidophenylalanine during each synthesis. The sequence used for analog 1 was the “Cantley Src peptide” (NH2-AEEEIYGEFEAKK-COOH), and the peptide sequence of analog 2 was the optimal Abl substrate (NH2-EAIYAAPFAKK-COOH) [ 58]. With the peptide still bound on the resin, the azidophenylalanine was reduced to aminophenylalanine by addition of a large excess of tributylphosphine in dimethylformamide. Bromoacetic acid was mixed with diisopropylcarbodiimide in a 2:1 molar ratio in dichloromethane. After stirring for 10 min the solvent was removed and the symmetric anhydride dissolved in dimethylformamide before addition to the resin. After cleavage from the resin the bromoacetylated peptide was precipitated in cold ether and lyophilized. The dried peptides were purified by reversed-phase HPLC (water:acetonitrile gradient) and confirmed by mass spectrometry. Coupling of the purified peptide to ATPγS was performed overnight with a small excess of ATPγS in 1 M ammonium acetate (pH 6.5). The substrate analogs were purified by gel filtration, dried, and stored at −80 °C. The structure of the compounds was verified by electrospray ionization mass spectrometry.

### Crystallography and refinement of Abl.

The substrate analogs were dissolved in buffer (100 mM Tris-HCl [pH 8.0] and 10 mM MgCl _2_) and added to the purified kinase domain of wild-type or H396P Abl in gel filtration buffer (50 mM Tris-HCl, 100 mM NaCl, 10% glycerol, 0.5 mM DTT). Crystals were grown in hanging drops with 10 mg/ml Abl kinase and 1 mM inhibitor. The crystals were cryoprotected in well solution plus 30% glycerol and frozen in liquid nitrogen. Diffraction data were collected at beamlines 8.2.2 and 12.3.1 of the Advanced Light Source.

Data were processed using Denzo and Scalepack [ 59] and molecular replacement was performed using the Phaser program [ 60] of the ccp4 suite. Refinement of molecular replacement solutions was performed using CNS [ 61], and atomic models were built with the program O [ 62].

The crystals of the wild-type protein ( Table 1, structure 1) grew in 0.2 M sodium acetate, 100 mM sodium cacodylate (pH 6.5), and 25% PEG 8,000 in space group P2 _1_ but had all unit cell angles equal to 90° (a = 62.6 Å, b = 78.4 Å, c = 141.6 Å). Given the orthorhombic unit cell and the monoclinic space group, diffraction data were susceptible to partial merohedral twinning. By screening X-ray data from many crystals we obtained a dataset to 1.8 Å resolution with negligible twinning. Refinement of the molecular model, obtained by molecular replacement using the Abl:PD173955 complex (PDB code 1M52), against these data produced satisfactory statistics (R-value of 21.9% and R _free_ = 24.4%), and deconvolution of twinned intensities during refinement was deemed unnecessary. The crystals contain four essentially identical molecules in the asymmetric unit (molecule B is a representative structure).

Crystals of the H396P mutant form of Abl in complex with substrate analog 1 ( Table 1, structure 2) grew in 2 M sodium malonate in space group P4 _1_2 _1_2 (a = 88.5 Å, b = 88.5 Å, c = 235.6 Å) but diffracted X-rays weakly. A molecular replacement solution was obtained with the Abl:PD173955 complex (PDB code 1M52) from which the activation loop had been deleted. Refinement of the model using group B factors to 3.1 Å resolution yielded interpretable electron density maps that clearly showed the presence of the inhibitor and the conformation of the activation loop. Some limited building was performed to fix regions of the structure clearly affected by the presence of the drug in the molecular replacement search model. The structure was refined to an R-value of 24.9% (R _free_ = 30.8%). The structure is very similar to the structure of Abl H396P bound to the small molecule inhibitor VX-680 [ 27].

Crystals of the kinase domain of Abl H396P in complex with bisubstrate inhibitor 2 ( Table 1, structure 3) were obtained in 0.1 M Bis-Tris (pH 5.5) and 25% PEG 3350 in space group P2 _1_2 _1_2 (a = 105.7 Å, b = 133.3 Å, c = 56.6 Å) and diffracted X-rays to 2.7 Å. The structure was solved by molecular replacement, as above, and refined to an R-value of 23.2% (R _free_ = 28.6%). The same crystal form (P2 _1_2 _1_2, a = 104.4 Å, b = 131.5 Å, c = 56.5 Å) was also obtained by cocrystallizing the kinase domain of Abl H396P with PD166326 (in 100% DMSO). These crystals grew in 0.1M citric acid (pH 3.5) and 25% PEG 3350. The crystals diffracted to higher resolution (2.0 Å), and the structure was refined to an R-value of 19.3% (R _free_ = 21.3%; Table 1, structure 4). The two models were refined independently but are almost identical. Significant differences are localized to the P-loop, the conformation of which depends on which ligand is bound to the kinase. These two P-loop conformations are the same as those previously visualized for complexes of Abl with PD166326 and for the structures of nucleotide complexes of protein kinases.

### Molecular dynamics.

Molecular dynamics trajectories were calculated using either the high-resolution structure of active, unphosphorylated Abl kinase domain [ 27], after the H396P mutation was mutated back to His, using the program O, or Src-like inactive Abl kinase domain (molecule B), as the starting points and the imatinib-bound structure, molecule A [ 9], as the target. All molecular dynamics simulations were performed with the TIP3P explicit water model [ 63] and with K ^+^ counterions. The initial structures were immersed in a truncated octahedral cell of water molecules and counterions were added using the LEAP module of AMBER, version 7, with the parm96 force field [ 64– 66]. The water extended at least 8 Å past the surface of the protein molecule. The SANDER module of AMBER was used to calculate the trajectories at constant temperature and pressure, SHAKE was used to constrain bonds to hydrogen [ 67], and the particle mesh Ewald summation method was used to calculate electrostatic interactions [ 68]. The system was initially equilibrated for 150 ps, and positional restraints were applied for the first 50 ps as described earlier [ 69]. In order to generate independent trajectories, different random-number seeds were used for the initial assignment of velocities, and this resulted in equilibrated structures with r.m.s. deviations of at least 1 Å from each other for all C _α_ atoms. Targeted molecular dynamics trajectories were calculated using SANDER [ 70]. D _T_ (see Equation 1 in the main text) was decremented continuously throughout the simulations, starting at the difference between the two crystal structures and ending at zero.

Eighty-six trajectories were calculated using different force constants ranging from 0.01 kcal mol ^−1^ Å ^−2^ to 100 kcal mol ^−1^ Å ^−2^. The trajectories spanned 200–225 ps when starting from the Src-like inactive structure and 200 ps when starting from the active structure, since the active structure was closer to the target in terms of r.m.s. deviation. Ten approximately 1-ns and two approximately 4-ns trajectories were also calculated. The entire protein was driven toward the target in some trajectories, whereas in some the restraint comprised the 25 residues in the activation loop. In most trajectories only 4–5 residues in the vicinity of the DFG motif were restrained. CHARMM was used to analyze the data [ 71]. In the simulations involving restraints on the whole kinase domain or the entire activation loop, the restraint set was used for the alignment of the simulation structure on the target structure. The alignment needed to be modified for the simulations involving only five or four residues, so as to provide a frame of reference for the DFG flip. In these simulations, the ten residues N-terminal to the DFG motif were also included in the alignments but not in the restraint potential. Equilibrium molecular dynamics simulations were set up as described above except that no targeting force was applied. These were used for comparison with the targeted molecular dynamics simulations and lasted at least 5 ns.

## Supporting Information

Dataset S1 Targeted Molecular Dynamics Trajectory following Transition 1 in Figure 1D
This dataset includes coordinates of all the atoms in the simulation sampled every several picoseconds and an example script for viewing the trajectory using PyMOL.
(16.5 MB ZIP)

Dataset S2 Targeted Molecular Dynamics Trajectory following Transition 2 in Figure 1D
This dataset includes coordinates of all the atoms in the simulation sampled every several picoseconds and an example script for viewing the trajectory using PyMOL.
(14.4 MB ZIP)

Figure S1 Sequence-Specific Interactions between the ATP–Peptide Conjugates and the Kinase Domain of Abl (A) The structure of the peptide–ATP conjugates. (B) Interactions between the ATP–peptide conjugate with the optimal peptide sequence and Abl molecule E. (C) Interactions between the ATP–peptide conjugate with the suboptimal peptide sequence and Abl molecule B.
(7.6 MB PDF)

Figure S2 Choice of Restraint Set and Force Constant for TMD (A) When only the residues ^379^VADF ^382^ are restrained, the deviation in the N-lobe is minimal. Gray: molecule B, starting structure; Yellow-orange: simulation final structure.
(B) Restraint force (pN), and r.m.s. distance of the restraint from the target (Å), plotted versus time (picoseconds) for two low-force constant (K = 0.15 kcal mol ^−1^ Å ^−2^) simulations starting with the active structure.
(2 MB PDF)

Figure S3 Targeted Molecular Dynamics Simulations Propose a Path for DFG Flipping (A) Coordination of Asp 381 during the DFG flip, starting from the Src-like inactive structure (molecule B) and following transition path B in backbone torsion angle space. The coordination of Asp 381 is almost identical for transition path A, except that the carbonyl of Asp 381 flips in the other direction.
(B) The side chain of Phe 382 passing through a hydrophobic pocket in the Src-like molecule for transition path B.
(C) The paths of Asp 381 and Phe 382 in backbone torsion angle space for two trajectories.
(17 MB PDF)

Figure S4 Molecule B Helps Explain Mutations in the Kinase Domain of Abl That Confer Resistance to Imatinib For two of these mutations we have shown the surface for all atoms within 6 Å of the mutated residue in the context of different structures. The active structure is shown in pink, the Abl:imatinib complex in green, and the Src-like structure (molecule B) in yellow-orange. (A) Phe 359. (B) Tyr 253.
(7.7 MB PDF)

Protocol S1 Basis for the Specificity of the Kinase Domain of Abl for Peptide Substrates (49 KB DOC)

Table S1 Crystallographic Data and Refinement (40 KB DOC)

Table S2 Hydrogen Bond Distances between Key Residues (26 KB DOC)

## Abbreviations

C-lobe: C-terminal lobe
DFG: Asp-Phe-Gly
HRD: His-Arg-Asp
H396P: His 396 Pro
N-lobe: N-terminal lobe
r.m.s.: root mean square
