# Supplementary material for: A Src-Like Inactive Conformation in the Abl Tyrosine Kinase Domain
Source: PLoS Biol. 2006 May 2;4(5):e144. doi: 10.1371/journal.pbio.0040144 (PMC1450098; doi:10.1371/journal.pbio.0040144)
Supplement: Supplementary file 1 [file pbio.0040144.sg001.pdf]

Figure S1

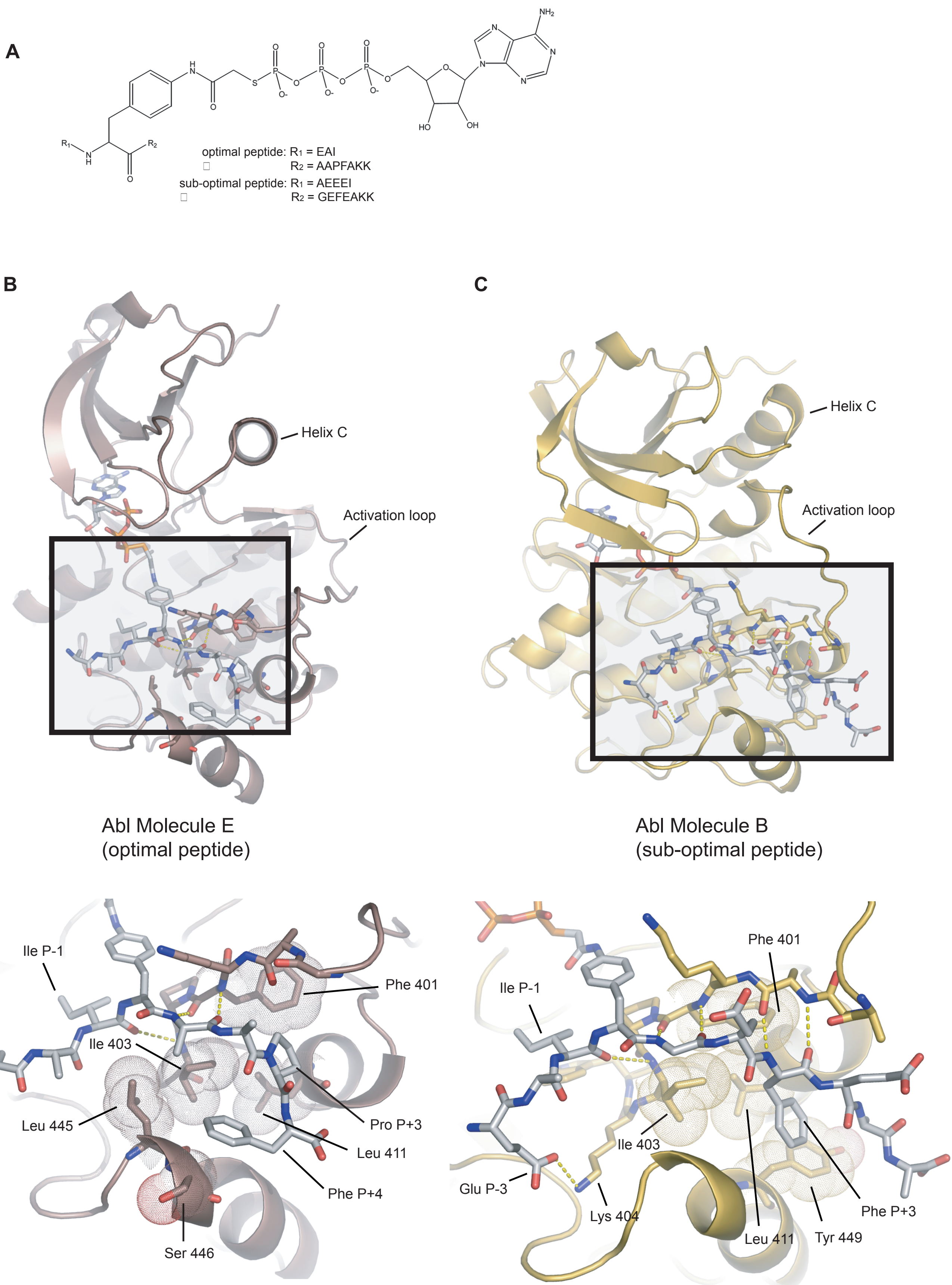
A
optimal peptide: R1 = EAI
R2 = AAPFAKK
sub-optimal peptide: R1 = AEEEI
R2 = GEFEAKK
B
C
Helix C
Activation loop
Helix C
Activation loop
Abl Molecule E
(optimal peptide)
Abl Molecule B
(sub-optimal peptide)
Ile P-1
Phe 401
Ile 403
Leu 445
Pro P+3
Leu 411
Phe P+4
Ser 446
Phe 401
Ile P-1
Ile 403
Glu P-3
Lys 404
Leu 411
Tyr 449
Phe P+3
