# Supplementary material for: A Src-Like Inactive Conformation in the Abl Tyrosine Kinase Domain
Source: PLoS Biol. 2006 May 2;4(5):e144. doi: 10.1371/journal.pbio.0040144 (PMC1450098; doi:10.1371/journal.pbio.0040144)

Figure S2

A

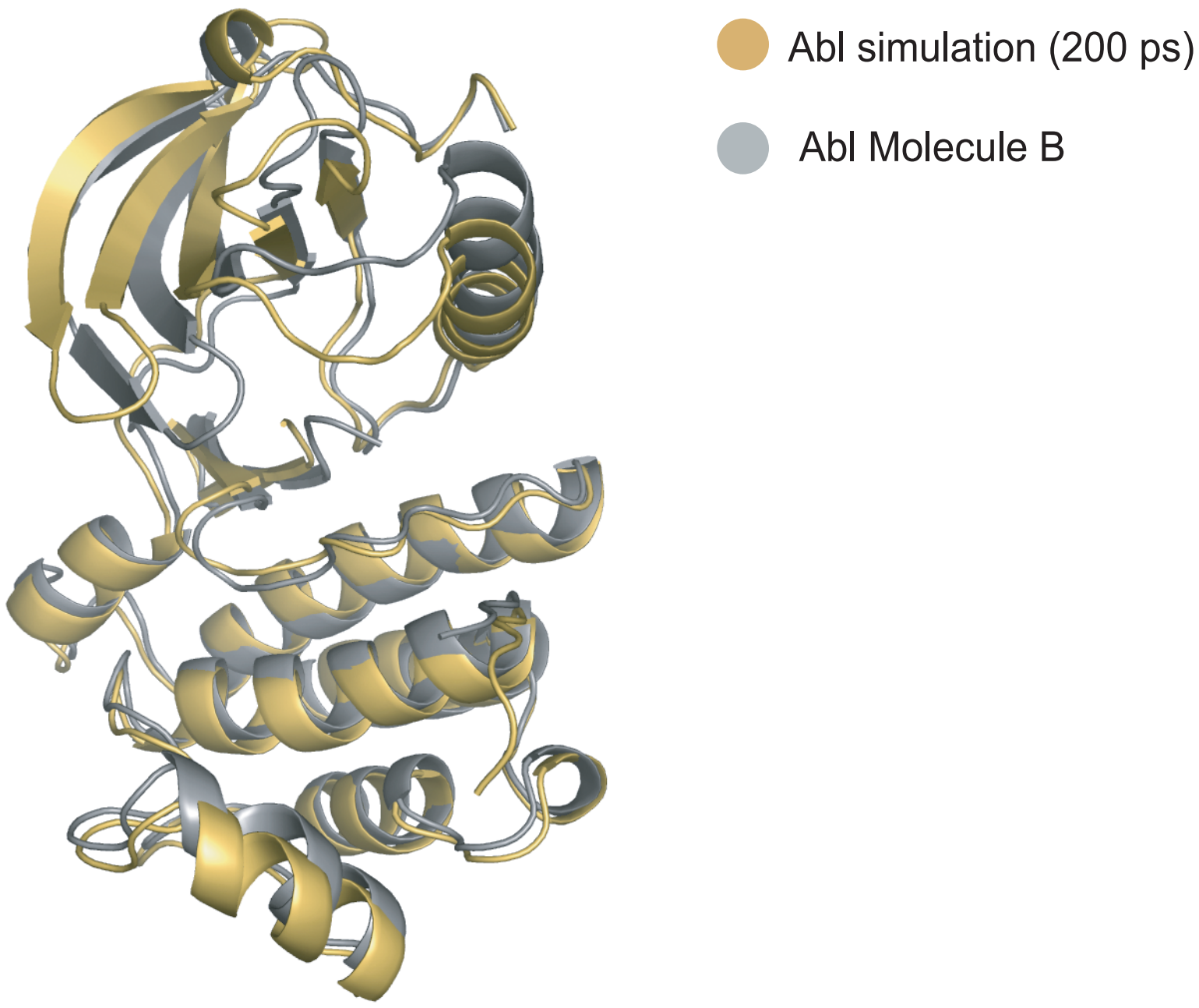

B From top to bottom: restraint force vs. time, r.m.s. deviation vs. time for two simulations (force constant K=10.5 pN/Å).

i) No DFG flip occurs

ii)Partial DFG flip occurs

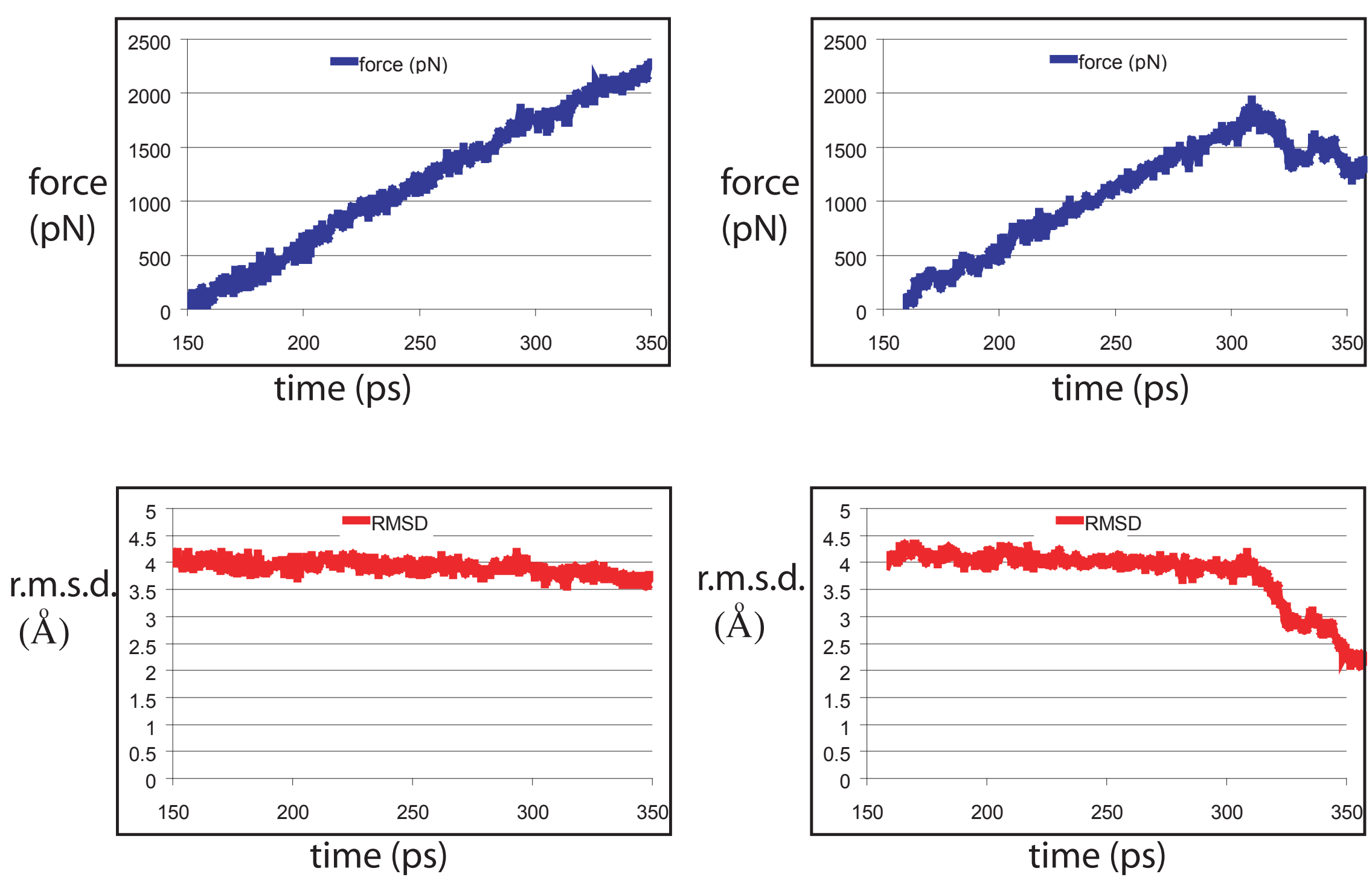

Supplement: Supplementary file 2 [file pbio.0040144.sg002.pdf]
