# Supplementary material for: A Src-Like Inactive Conformation in the Abl Tyrosine Kinase Domain
Source: PLoS Biol. 2006 May 2;4(5):e144. doi: 10.1371/journal.pbio.0040144 (PMC1450098; doi:10.1371/journal.pbio.0040144)
Supplement: Supplementary file 3 [file pbio.0040144.sg003.pdf]

Figure S3

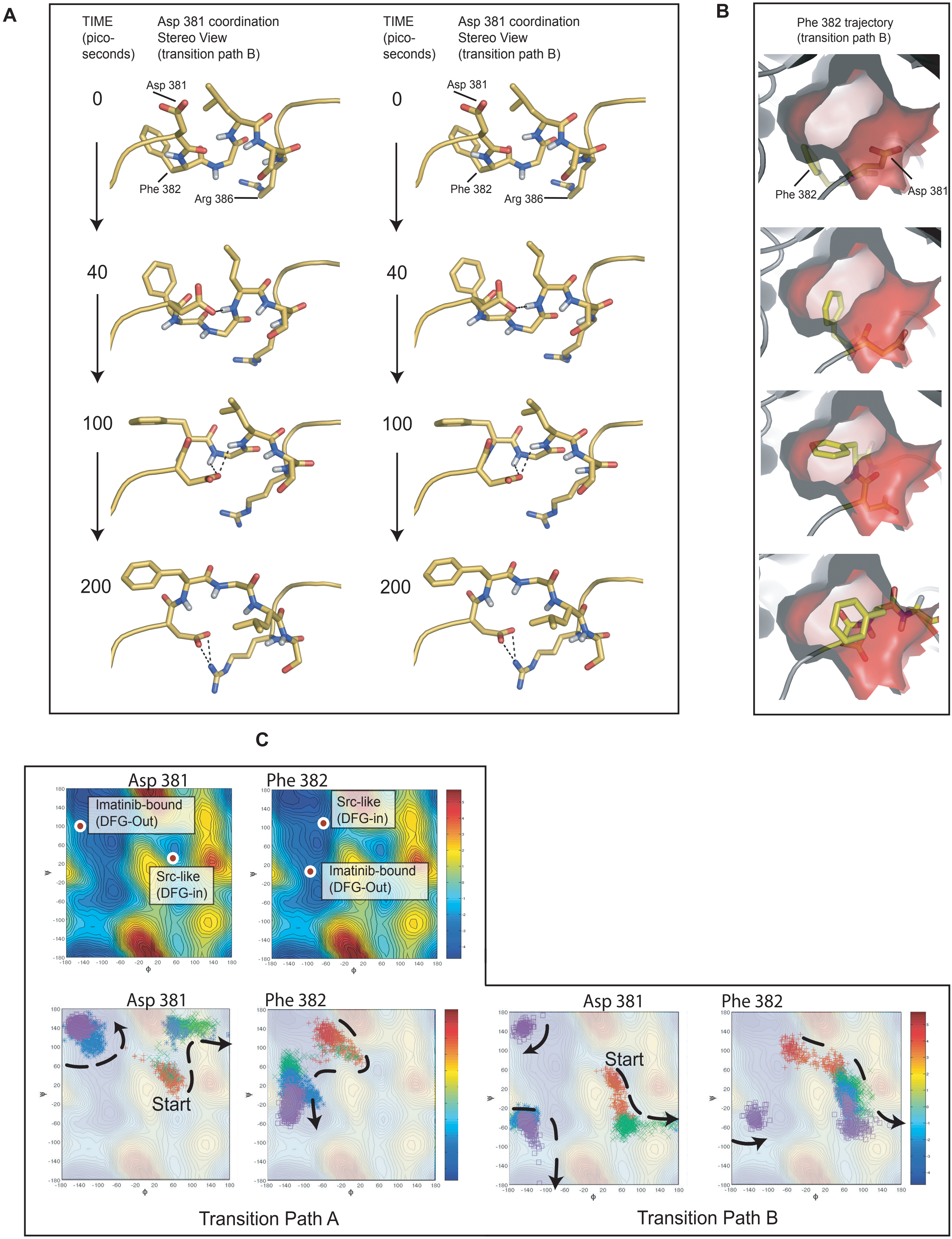
A
TIME (pico-seconds)
Asp 381 coordination Stereo View (transition path B)
TIME (pico-seconds)
Asp 381 coordination Stereo View (transition path B)
0
40
100
200
Asp 381
Phe 382
Arg 386
B
Phe 382 trajectory (transition path B)
Phe 382
Asp 381
C
Asp 381
Imatinib-bound (DFG-Out)
Src-like (DFG-in)
Phe 382
Src-like (DFG-in)
Imatinib-bound (DFG-Out)
Start
Transition Path A
Transition Path B
