# Supplementary material for: A Src-Like Inactive Conformation in the Abl Tyrosine Kinase Domain
Source: PLoS Biol. 2006 May 2;4(5):e144. doi: 10.1371/journal.pbio.0040144 (PMC1450098; doi:10.1371/journal.pbio.0040144)
Supplement: Supplementary file 4 [file pbio.0040144.sg004.pdf]

Figure S4

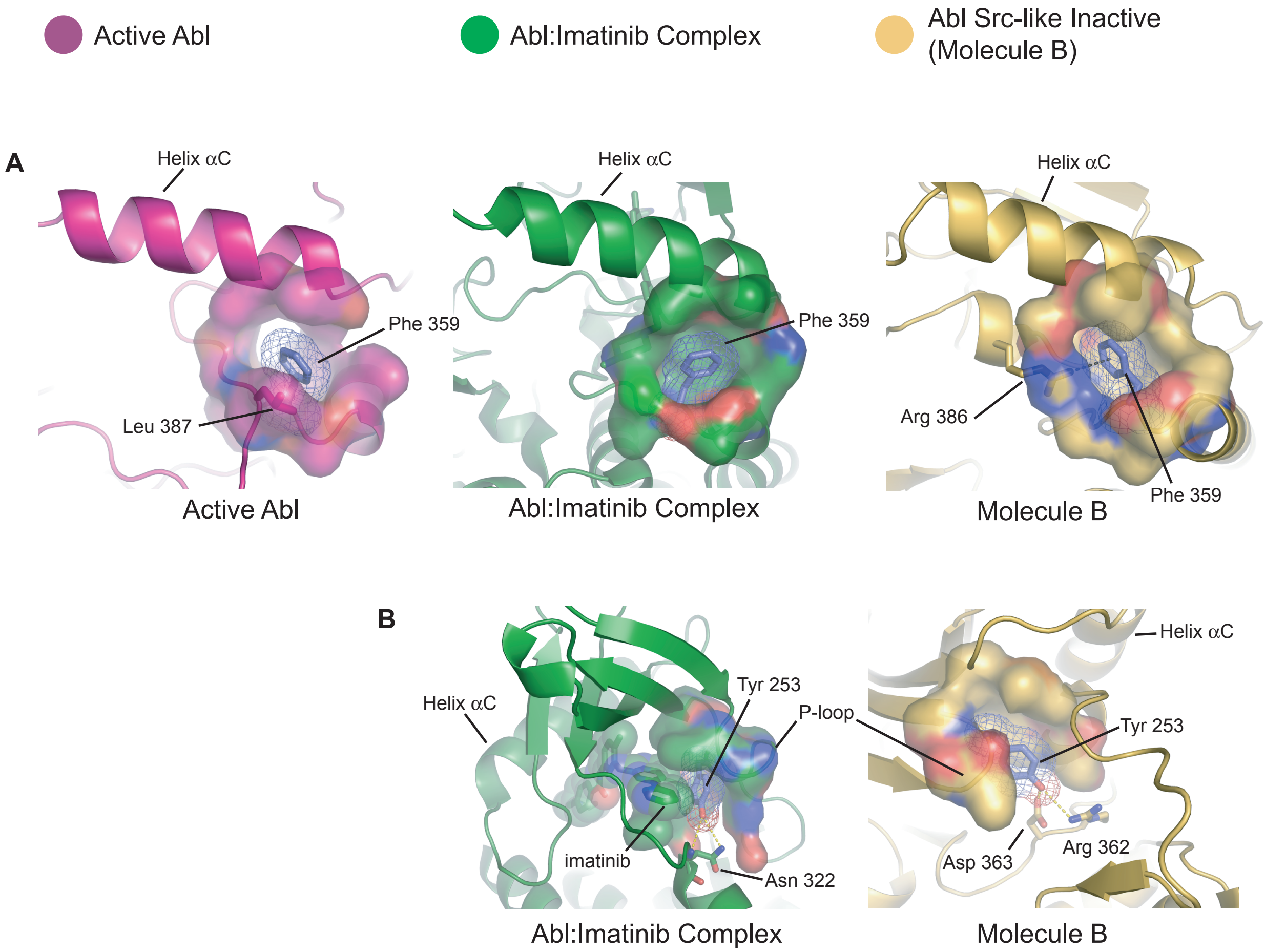

Active Abl
Abl:Imatinib Complex
Abl Src-like Inactive (Molecule B)
A
Helix αC
Phe 359
Leu 387
Active Abl
Helix αC
Phe 359
Abl:Imatinib Complex
Helix αC
Arg 386
Phe 359
Molecule B
B
Helix αC
Tyr 253
P-loop
imatinib
Asn 322
Abl:Imatinib Complex
Helix αC
Tyr 253
Asp 363
Arg 362
Molecule B
