# Supplementary material for: A Src-Like Inactive Conformation in the Abl Tyrosine Kinase Domain
Source: PLoS Biol. 2006 May 2;4(5):e144. doi: 10.1371/journal.pbio.0040144 (PMC1450098; doi:10.1371/journal.pbio.0040144)
Supplement: Supplementary file 7 [file pbio.0040144.sd003.doc]

# Protocol S1: Supporting Information

## Basis for the specificity of the kinase domain of Abl for peptide substrates

The crystal structures reported in this work were obtained using two different ATP analog-peptide conjugates that differed in the sequence of the peptide constituent (see Experimental Procedures and Figure S1). The analysis of the interactions between the peptides and the kinase is complicated by the fact that the protein adopts different conformations in the different crystal structures. However, in the structures solved using the sub-optimal peptide sequence (Structures 1 and 2, Table 1) the peptide is observed to bind in the same manner. We are therefore confident that the binding mode is an intrinsic property of the peptide sequence and not simply a consequence of the local conformation of the kinase.

In all three cases, the central portion of the peptide flanking the tyrosine derivative is ordered and binds to the C-terminal lobe of the kinase. The overall mode of binding is the same in the three structures, with the formation of an anti-parallel β-sheet between the peptide and the C-terminal portion of the activation loop. This mode of binding was first demonstrated for substrates of the receptor tyrosine kinase Irk [1]. The sequences of both peptides contain a glutamate at P-3 and an isoleucine at P-1. The glutamate residue forms an ion pair with Lys 404 in the C-terminal lobe of the kinase, while the isoleucine sidechain makes hydrophobic contacts with Trp 405 and Pro 402.

There are clear differences between the binding of the optimal- and sub-optimal peptides (Figure S1). In the case of the optimal peptide, the sidechains of proline (P+3) and phenylalanine (P+4) fit snugly into a hydrophobic pocket in the C-lobe of the kinase. The peptide with the sub-optimal sequence follows a slightly different path in this region of the structure which positions the sidechain of phenylalanine (P+3) in the same pocket, burying considerably less hydrophobic surface area. This may account for the differences in the efficiencies with which the two peptides are phosphorylated by Abl [2]. The observation that the peptide backbone adjusts depending on the location of the phenylalanine sidechain (P+3 in the sub-optimal peptide and P+4 in the optimal peptide) suggests that hydrophobic interactions may dominate the binding of the substrate peptides. The relative paucity of sequence-specific interactions is consistent with experimental data reporting that Abl is a relatively promiscuous kinase. Given that these two peptide-ATP conjugates inhibit Abl with a very similar potency (0.95 μM and 1.2 μM for the optimal and sub-optimal conjugates, respectively), it is likely that the peptide interactions make only a minor energetic contribution to the affinity of these compounds.

The variation in the path followed by the peptide backbone of the two different substrate peptides indicates that a change in substrate sequence may result in a somewhat altered mode of binding. This emphasizes that care must be taken in attributing kinase specificity to interactions observed in a single crystal structure.

## Presence of Nucleotide and Mg^2+^ in targeted molecular dynamics simulations

Nucleotide and Mg^2+^ were not included in most simulations. Two simulations of transition 2 (Figure 1d) with ATP•Mg^2+^ or ADP•Mg^2+^ bound indicated that although the general features of the transitions are similar to those seen in simulations without nucleotide, increased forces are required to break the Asp-Mg^2+^ bond. It is not clear whether the DFG flip occurs in the presence of bound nucleotide or in transient states when nucleotide is released, and our calculations do not address this issue.

## Restraint sets and force constants in targeted molecular dynamics simulations

Multiple simulations were carried out using restraint sets that included the entire kinase domain, the kinase domain except residues 383-438 in the activation loop, the entire activation loop (residues 341-438), five residues including the DFG motif (^379^VADFG^383^) or four residues including only the aspartate and phenylalanine residues of the DFG motif (^379^VADF^382^).

The conformation of the activation loop is significantly different in the active, the Src-like inactive and in the imatinib-bound conformations (Figure 1). The inclusion of the entire activation loop in the restraint leads to collisions with the N-lobe of the kinase, causing unreasonably large structural excursions. Most likely, the activation loop has to first unfold, and then refold in both transitions 1 and 2 (Figure 1d), and our restraint strategy does not capture such a stepwise process. Restricting the restraint set to just the DFG motif and the adjacent residues allows the transition to occur with minimal adjustment to the rest of the structure (Figure S2a). These considerations lead us to focus most of our analysis on simulations in which the restraint force was applied to the four residue segment ^379^VADF^382^.

We carried out simulations with force constants ranging from 10 kcal mol^-1^ Å^-2^ (69.7 × 10^3^ pN atom^-1^ Å^-1^) to 0.01 kcal mol^-1^ Å^-2^ (976 pN atom^-1^ Å^-1^). Using equation (1), we can estimate the extent to which the simulation structure can lag behind the driving force due to thermal energy by calculating the value of $\left|D-D_{T}\right|$ that corresponds to 1 k_B_T of energy. For 58 atoms in the restraint set (N=58), $\left|D-D_{T}\right|$is ~0.1 Å when k is 10 kcal mol^-1^ Å^-2^, and the transition proceeds smoothly because the simulation tracks the restraint closely. For a force constant of 0.15 kcal mol^-1^ Å^-2^, $\left|D-D_{T}\right|$is ~0.8 Å, and the transition is discontinuous because the simulated structure lags behind the target until the forces build up to the point where a transition occurs (Figure S2b). When the force constant is reduced below 0.15 kcal mol^-1^ Å^-2^, the transition no longer occurs in 200-225 ps.

The simulations with the lowest force constant for which DFG flips are seen to occur (0.15 kcal mol^-1^ Å^-2^) correspond to very high driving forces (Figure S2b). For example, in one such simulation the DFG motif does not begin to flip until the force builds up to approximately 2000 pN. This value of the force is much larger than the experimentally measured force of ~160 pN required to pull biotin away from streptavidin, or the stall forces of the strongest known biological motors (50-65 pN) [3,4]. The large forces in the simulation are probably a consequence of the short trajectory lengths, which do not allow full relaxation of the rest of the protein structure.

## Further Analysis of Imatinib-Resistance Mutations in Abl

In addition to the mutations discussed in the main text, two other imatinib resistance mutations appear to preferentially destabilize the Src-like inactive structure of Abl (molecule B). Mutation of Phe 359 to cysteine, valine or aspartate (F359C/V/D) has been seen in CML patients who have ceased to respond to imatinib [5], and the F359C mutation was also isolated in the in-vitro screen [6]. The C_β_ atom of Phe 359 makes peripheral contact with imatinib in the drug complex (Figure S4a, center), and the remainder of the sidechain points out into solvent. In the active structure of Abl the phenyl ring of Phe 359 packs against Leu 387 in the activation loop (Figure S4a, left) such that mutations at this position might be expected to interfere with the active conformation and to sensitize the protein to imatinib rather than cause resistance. In contrast, Phe 359 plays a more striking structural role in the Src-like inactive conformation of Abl. The sidechain makes extensive hydrophobic contacts in the rearranged interface between the activation loop and helix αC (Figure S4a, right). In addition, an amino-aromatic interaction occurs between the phenyl ring of Phe 359 and one of the sidechain amino groups of Arg 386.

Tyrosine 253 is a residue in the phosphate binding P-loop that is mutated to either Phe or His in patients. The conformation of the P-loop is important for imatinib binding and several other resistance mutations map to this region. In the case of Tyr 253, calorimetric measurements indicate that imatinib binding is not affected by the mutations (M. Nidanie Henderson, personal communication). In the Src-like inactive structure Tyr 253 forms hydrogen bonds to Asp 363 and Arg 362, and mutation to Phe or His would disrupt these interactions (Figure S4b).

## Kinetic characterization of peptide-ATP conjugates

The potency of the peptide-ATP conjugates as inhibitors of Abl was assessed using a spectrophotometric kinase assay performed with the wild-type kinase domain of Abl[7]. Peptide and ATP substrates were present at 100 μM and 50 μM concentrations, respectively. By assuming that the peptide-ATP conjugates are linear competitive inhibitors of both ATP and peptide substrates the IC_50_ can be related to the K_i_ by the following procedure:
1) IC_50_ = K_i,apparent_ (1 + A/K_m_(A))

2) K_i,apparent_ = K_i_(1 + B/K_m_(B))

where A and B are the concentrations of ATP and peptide substrates, respectively, used in the assay. The K_m_(A) and K­_m_(B) values are the K_m_ values for these substrates which were determined using the same spectrophotometric assay used for the IC_50_ experiments.

The K_m_ values for peptide and ATP are 70 and 21 μM, respectively, under the conditions of the assay. The K_i_ values for the Abl-optimal and sub-optimal peptide-ATP conjugates (see Experimental Procedures and Figure S1) were 0.95 μM and 1.2 μM, respectively.

# Supplementary Tables

**Table S1.** Crystallographic Data and Refinement

**Table S2.** Hydrogen bond distances between key residues

# Supplementary Figures

**Figure S1. Sequence-specific interactions between the ATP-peptide conjugates and the kinase domain of Abl.** **A)** The structure of the peptide-ATP conjugates. **B)** Interactions between the ATP-peptide conjugate with the optimal peptide sequence and Abl molecule D. **C)** Interactions between the ATP-peptide conjugate with the sub-optimal peptide sequence and Abl molecule B.

**Figure S2. Choice of restraint set and force constant for TMD.** **A)** When only the residues ^379^VADF^382^ are restrained, the deviation in the N-lobe is minimal. Grey: molecule B, starting structure; Yellow-orange: simulation final structure **B)** Restraint force (pN), and r.m.s. distance of the restraint from the target (Å), plotted versus time (picoseconds) for two low force constant (K=0.15 kcal mol^-1^ Å^-2^) simulations starting with the active structure.

**Figure S3. Targeted Molecular Dynamics simulations propose a path for DFG flipping. A)** Coordination of Asp 381 during the DFG flip, starting from the Src-like inactive structure (molecule B) and following transition path B in backbone torsion angle space. The coordination of Asp 381 is almost identical for transition path A, except that the carbonyl of Asp 381 flips in the other direction **B)** The sidechain of Phe 382 passing through a hydrophobic pocket in the Src-like molecule for transition path B **C­)** The paths of Asp 381 and Phe 382 in backbone torsion angle space for two trajectories.

**Figure S4.** **Molecule B helps explain mutations in the kinase domain of Abl that confer resistance to imatinib.** For two of these mutations we have shown the surface for all atoms within 6 Å of the mutated residue in the context of different structures. The active structure is shown in pink, the Abl:imatinib complex in green and the Src-like structure (molecule B) in yellow-orange. **A)** Phe 359 **B)** Tyr 253

# Supplementary References

1. Hubbard SR (1997) Crystal structure of the activated insulin receptor tyrosine kinase in complex with peptide substrate and ATP analog. Embo J 16: 5572-5581.

2. Songyang Z, Carraway KL, 3rd, Eck MJ, Harrison SC, Feldman RA, et al. (1995) Catalytic specificity of protein-tyrosine kinases is critical for selective signalling. Nature 373: 536-539.

3. Isralewitz B, Gao M, Schulten K (2001) Steered molecular dynamics and mechanical functions of proteins. Curr Opin Struct Biol 11: 224-230.

4. Wang HY, Elston T, Mogilner A, Oster G (1998) Force generation in RNA polymerase. Biophys J 74: 1186-1202.

5. Shah NP, Nicoll JM, Nagar B, Gorre ME, Paquette RL, et al. (2002) Multiple BCR-ABL kinase domain mutations confer polyclonal resistance to the tyrosine kinase inhibitor imatinib (STI571) in chronic phase and blast crisis chronic myeloid leukemia. Cancer Cell 2: 117-125.

6. Azam M, Latek RR, Daley GQ (2003) Mechanisms of autoinhibition and STI-571/imatinib resistance revealed by mutagenesis of BCR-ABL. Cell 112: 831-843.

7. Seeliger MA, Young M, Henderson MN, Pellicena P, King DS, et al. (2005) High yield bacterial expression of active c-Abl and c-Src tyrosine kinases. Protein Sci 14: 3135-3139.
