# Supplementary material for: A Src-Like Inactive Conformation in the Abl Tyrosine Kinase Domain
Source: PLoS Biol. 2006 May 2;4(5):e144. doi: 10.1371/journal.pbio.0040144 (PMC1450098; doi:10.1371/journal.pbio.0040144)
Supplement: Supplementary file 8 [file pbio.0040144.st001.doc]

**Table S1. Crystallographic Data and Refinement**

|  |  | Structure 1  PDB ID: 2G1T | Structure 2  PDB ID: 2G2I | Structure 3  PDB ID: 2G2F | Structure 4  PDB ID: 2G2H |
| --- | --- | --- | --- | --- | --- |
|  | Space Group | P2_1_ | P4_1_2_1_2 | P2_1_2_1_2 | P2_1_2_1_2 |
|  | Unit Cell  (all angles are 90°) | a = 62.6 Å | a = 88.5 Å | a = 105.7 Å | a = 104.4 Å |
|  |  | b = 78.4 Å | b = 88.5 Å | b = 133.3 Å | b = 131.5 Å |
|  |  | c = 141.6 Å | c = 235.6 Å | c = 56.6 Å | c = 56.5 Å |
| Data Collection | Resolution  Range (Å) | 50-1.8 | 50-3.1 | 50-2.7 | 50-2.0 |
|  | Unique Reflections | 117589 | 18261 | 27408 | 79736 |
|  | Completeness^a^ (%) | 97.8 (85.3) | 99.3 (95.1) | 99.8 (98.3) | 99.5 (99.8) |
| Model Refinement | I/σ^a^ | 10.8 (2.15) | 14.2 (2.75) | 9.54 (2.18) | 11.04 (3.99) |
|  | R_sym_^a,b^ (%) | 6.7 (43.7) | 9.9 (44.5) | 7.8 (48.0) | 4.0 (41.4) |
|  | Protein Molecules | 4 | 2 | 2 | 2 |
|  | Water Molecules | 902 | 0 | 82 | 414 |
|  | R_work_^c^ / R_free_ ^d^(%) | 21.9 / 24.4 | 24.9 / 30.8 | 23.2 / 28.6 | 19.3 / 21.3 |

^a^ Number in parentheses is for highest resolution shell.

^b^ $R_{sym}=\frac{\sum\left|I_{h}-\left\langle I_{h}\right\rangle\right|}{\sum I_{h}}$, where $\left\langle I_{h}\right\rangle$is the average intensity over symmetry equivalent reflections

^c^	$R_{work}=\frac{\sum\left|F_{obs}-F_{calc}\right|}{\sum F_{obs}}$, where the summation is over all data used in refinement

^d^ R_free_ was calculated using the 10% of reflection data that were excluded during crystallographic refinement
