# Supplementary material for: A Src-Like Inactive Conformation in the Abl Tyrosine Kinase Domain
Source: PLoS Biol. 2006 May 2;4(5):e144. doi: 10.1371/journal.pbio.0040144 (PMC1450098; doi:10.1371/journal.pbio.0040144)
Supplement: Supplementary file 9 [file pbio.0040144.st002.doc]

**Table S2.** Hydrogen bond distances between key residues

| Structure | Interaction | Hydrogen bond length  (Å) |
| --- | --- | --- |
| Src-like Inactive  Table 1: Molecule B, Struct. 1 | Glu 286-Arg 386 | 3.08 |
|  | Asp 381-Lys 271 | 3.31 |
| Active Abl  Table 1: Molecule C, Struct. 2 | Glu 286-Lys 271 | 3.25 |
| Intermediate Abl  Table 1: Molecule E, Struct. 3 | Glu 286-Arg 362 | 2.77 |
| Intermediate Abl  Table 1: Molecule G, Struct. 4 | Glu 286-Arg 362 | 2.94 |
